# Sign Language Ability in Young Deaf Signers Predicts Comprehension of Written Sentences in English

**DOI:** 10.1371/journal.pone.0089994

**Published:** 2014-02-28

**Authors:** Kathy N. Andrew, Jennifer Hoshooley, Marc F. Joanisse

**Affiliations:** Department of Psychology and Brain and Mind Institute, The University of Western Ontario, London, Ontario, Canada; Birkbeck College, United Kingdom

## Abstract

We investigated the robust correlation between American Sign Language (ASL) and English reading ability in 51 young deaf signers ages 7;3 to 19;0. Signers were divided into ‘skilled’ and ‘less-skilled’ signer groups based on their performance on three measures of ASL. We next assessed reading comprehension of four English sentence structures (actives, passives, pronouns, reflexive pronouns) using a sentence-to-picture-matching task. Of interest was the extent to which ASL proficiency provided a foundation for lexical and syntactic processes of English. Skilled signers outperformed less-skilled signers overall. Error analyses further indicated greater single-word recognition difficulties in less-skilled signers marked by a higher rate of errors reflecting an inability to identify the actors and actions described in the sentence. Our findings provide evidence that increased ASL ability supports English sentence comprehension both at the levels of individual words and syntax. This is consistent with the theory that first language learning promotes second language through transference of linguistic elements irrespective of the transparency of mapping of grammatical structures between the two languages.

## Introduction

Learning to read is a difficult task for most deaf individuals. Sign languages like American Sign Language (ASL) do not have a written form, and a written language like English is not an orthographic code for a signed language. Thus, a deaf individual who is learning to read is literally learning a foreign language through a modality that is only partially accessible (i.e., through orthography but not phonology).

One of the principal explanations for reading difficulties in deaf signers is their lack of access to the phonological code of a written language [1]–[2]. Hearing individuals learn a spoken language through the auditory modality prior to learning to read, and subsequently learn to map the written word onto their knowledge of this spoken code. Thus, when a hearing reader sees a word on the page they have access to the word's orthographic form and, at least in alphabetic orthographies like English, can compute its phonological form. This access to phonology allows hearing readers to parse written words into their individual phonemes making the semantics of even unfamiliar words accessible through decomposition. Deaf readers on the other hand must rely more heavily on orthography to access meaning in written language (notwithstanding evidence suggesting that some higher-achieving deaf readers have some limited access to phonology [3]–[5]).

Although learning to read is difficult for all deaf individuals, there can be significant variation in this respect, and for reasons that are not always transparent. That said, degree of ASL proficiency has been repeatedly shown to be the single best predictor of English reading outcomes in the deaf population (for a review and meta-analysis, see [6]). For instance, Strong and Prinz [7] compared ASL signers with low, medium and high signing ability on English proficiency, where proficiency in either language was operationalized as a composite score of a variety of comprehension and production tasks. They found that ASL skill was significantly correlated with English ability, such that the high ability group outperformed both the medium and low ability groups, and the medium ability group outperformed the lowest ability group. The authors present this finding as evidence that increased levels of ASL ability lead to increased English proficiency. Padden and Ramsey [8] drew similar conclusions defining English ability via a collection of subtasks from the Stanford Achievement Test (adapted for deaf participants).

Several others have reported similar findings, where ASL proficiency is correlated with English reading outcomes, both in children [9]–[11] and adults [12]. However these studies all examined the relationship between ASL and English in a relatively broad sense, using general comprehension and production tasks spanning a range of language abilities.

In contrast, studies have not tended to investigate this issue with regard to finer-grained aspects of language processing. An exception here is Mayberry and Lock [13], who assessed performance on specific English sentence constructions including a passive sentence construction, and found that deaf adults who had early language exposure performed similarly to native English speakers, whereas deaf adults without early language exposure performed more poorly on this construction.

The present study takes a closer look at English syntactic comprehension in deaf signers of ASL, with regard both to individuals' comprehension accuracy as well as the types of errors produced by both deaf individuals and hearing native English speakers. Our goal was to investigate in a more detailed manner whether ASL proficiency predicts learning specific elements of English grammar, or whether it is limited to a more basic transfer like vocabulary learning.

This investigation builds on two models of second language learning that focus on the role of first language (L1) proficiency in determining second language (L2) outcomes. Cummins' Linguistic Interdependence Hypothesis [14] proposes that L2 learning is dependent on the degree to which the individual has learned L1 before extensive exposure to L2 begins. Accordingly, for primarily unilingual children to effectively learn L2, it is essential that a concretely link first be established between real world concepts and L1 lexical items. MacWhinney's Unified Competition Model [15] represents a more comprehensive view of this relationship where not only lexical items transfer between languages. Elaborating on the earlier Competition Model of learning [16], it proposes that initially, learners infer that L2 properties map directly onto L1, regardless of actual fit, and that this occurs across multiple levels of linguistic analysis including lexical knowledge but also sensory processing, and sentence comprehension (described by MacWhinney as: ‘grammatical role decoding’ plus ‘comprehension’). For the purposes of our study, we focus on the lexical and sentence comprehension levels since there is no clear mechanism by which one can gauge the sensory processing similarities of an ASL sign and a printed English word.

Transference in lexical knowledge takes place when there is high conceptual overlap between lexical items in L1 and L2. ASL learners should find it easier to comprehend English words when they can be directly mapped onto known signs. In both Cummins' and MacWhinney's models, L1 acts as an intermediary between the lexical item in L2 and the concept itself. This raises the first question of the present study: is the relationship between L1 and L2 learning in ASL strictly due to better signers having larger vocabularies, and thus being better able to map known concepts onto written English words?

The alternative is that L2 facilitation occurs at both the lexical and sentence comprehension (syntactic) levels. The task of understanding a full sentence goes well beyond assigning meaning to the individual words in a sentence. In order to extract the full meaning of the sentence, readers must also understand the language's syntactic constructions. According to the Unified Competition Model successful transference of L2 syntactic structures to L1 depends on the degree of syntactic match between those structures. Transference in sentence comprehension can thus be described as the extent to which syntactic principles of L1 reflect syntactic principles in L2.

As it turns out, English syntactic constructions vary with respect to how transparently they map onto ASL constructions. This permits us to examine whether the relative similarity of an ASL and English construction affects signers' ability to learn the construction in English. In this study we addressed this via four sentence types, active, passive, pronoun, reflexive pronoun, which map between languages to differing degrees. We group these sentence types into two separate syntactic investigations: pronoun binding trials (pronouns, reflexives) and word order trials (actives, passives).

Non-reflexive and reflexive pronouns exist in both ASL and English: while ASL uses spatial cues, inflections on agreeing verbs and even eye gaze to bind a pronoun or reflexive to its antecedent, in English this is achieved through a syntactic relationship. In English, non-reflexive pronouns refer back to an earlier antecedent that is indicated by a different word at an earlier point in a sentence (e.g., *him* refers back to *John* in “John said that Alice likes him”). Sometimes understanding who is being referred to by the pronoun is non-ambiguous, as in the example above. Other times, the relationship is more ambiguous (e.g., *him* refers back to *John* in “John said that Jack likes him”). In contrast, ASL pronoun use is accomplished by indexing the exact location in which the entity was previously set up in physical space. Since all possible referents occupy their own unique position in the signing space, identifying the antecedent of a pronoun is non-ambiguous in ASL [17]. As such, we propose that non-reflexive English pronoun sentences may be difficult for signers due to the potential for ambiguity in these constructions, which does not map clearly onto ASL pronouns.

Reflexive pronouns also exist in both English and ASL. In English they take the form, *herself*, *itself* etc., always ending in the marker, *self*. ASL employs a specific handshape (the ‘A’ handshape) that indicates the idea of SELFNESS when articulated in the direction of the referent's spatial location as established by the signer, (e.g., HIMSELF, MYSELF). Thus reflexives are produced similarly in the two languages, both using a specific marker (*self* in the case of English, and the *A* handshape in the case of ASL) to clearly identify the reflexive pronoun. We expect that these sentences will be less difficult for signers than the non-reflexive pronouns because the mapping between languages is more straightforward.

The most prominent description of the structure of pronouns comes from Chomsky's Binding Theory [18]. The theory explains why *mother*, but not *girl*, is an acceptable antecedent to *herself* in the reflexive pronoun sentence, “The girl says the mother washes herself”, and why *father*, but not *boy*, is an acceptable antecedent to *him* in the non-reflexive pronoun sentence, “The father says the boy pushed him.” Previous research suggests that children typically acquire comprehension of reflexives earlier and more consistently than non-reflexives [19]–[21].

The second syntactic manipulation of interest in this study involved the active/passive alternation in word order. This is of interest since ASL permits some freedom in choice of word order. However, the principle of economization demands clarity and efficiency in ASL [17] such that signers tend to focus production on content, exclude function words, and minimize the use of word orders that add unnecessary lexical items and ambiguity to utterances. Indeed in both English and ASL the most typical way to express a transitive relationship is S-V-O [22]. Note that there are reports of passive-like constructions in ASL, where a signer takes on the role of the object of the sentence, thereby shifting focus toward the object. For instance, Janzen et al. [23] suggest the example of “a girl punching a boy.” In order to explain what is happening in the picture, the signer might use the location of signs relative to their own body position in order to set up the girl on the right (with body facing slightly left) and the boy on the left (with body facing slightly right). The signer then reassumes the position of the girl and produces a punching motion in the direction of the boy, then quickly switches *to become the body of the boy while the signer's oncoming fist is understood to be that of the girl's*. The italicized portion of the above sentence where the boy is looking towards the oncoming fist from the direction of the girl might be taken as an equivalent to the English passive: “the boy was punched by the girl”. While this shift in focus is what we consider to be a passive in English, when it is used in ASL there is much set up before the production of the passive-like formation. That is, the passive-like construction does not stand alone as a full sentence but rather is part of the sentence that serves to emphasize focus on the object at a particular point within the sentence. A true ASL equivalent of the English passive construction is thus difficult to positively identify and has gone largely unmentioned in studies of ASL acquisition. Since it does not compare well to English passives it seems reasonable to assume that the mapping between the constructions (if ASL does in fact have such a construction) in the two languages is non-obvious at best.

There is good evidence that hearing children acquire passives later than active sentences [24] especially ‘reversible’ passives (e.g., “The mother was hugged by the daughter”) where the subject and object can only be inferred through syntactic means. There is also some evidence that deaf signers perform poorly on passives relative to same age hearing children, though less is known as to whether this is due to a general difficulty in word recognition, or a more specific difficulty with processing English syntactic structures [25].

### Rationale of present study

We examined how general ASL proficiency relates to increased English sentence comprehension in school-age deaf signers. The Linguistic Interdependence Hypothesis and the Unified Competition Model both predict that skilled signers should show better reading outcomes than less-skilled signers because increased ASL ability should facilitate English reading ability in a concrete manner: transference of lexical items should allow for comprehension of individual English words. Further, the Unified Competition Model predicts that facilitation should also occur at the level of sentence comprehension, such that more advanced ASL signers should have improved ability to decode English syntax. We examined the key assumption in both theories, that L2 proficiency reflects L1 proficiency, as well as the ways in which L1 proficiency constrains L2 learning with the Unified Competition Model in mind. Of interest was distinguishing advantages due to improved single word reading skill from more advanced syntactic comprehension strategies.

In order to investigate these questions, participants performed an assessment of ASL abilities (which included vocabulary, sign decision, and story comprehension measures) and an English sentence comprehension task that spanned four sentence structures (active, passive, pronoun and reflexive). Participants were divided into two groups based on signing achievement, and English sentence comprehension (accuracy and error tendencies) was compared across groups. We expected to see increased syntactic processing ability by skilled signers relative to less-skilled signers marked by increased accuracy on across the syntactic structures. This finding would be consistent with the Linguistic Interdependence Hypothesis where second language acquisition reflects first language proficiency. Additionally, we expected less-skilled signers to make both word recognition and syntactic parsing errors, but skilled signers to tend towards syntactic parsing errors only. This finding would be consistent with the Unified Competition Model such that even when syntax becomes an obstacle less-skilled signers continue to use their lexical knowledge to approach the correct answer. Further in line with the Unified Competition Model, we expected passive sentences to be particularly difficult for all deaf readers, since there is no obvious analogue in ASL, and exposure to English passives is limited in the deaf population [26]. While both the Linguistic Interdependence Hypothesis and the Unified Competition Model were conceived of to explain L2 learning in relation to L1 proficiency in spoken languages, here the models are used in a novel fashion—to explain L2 learning of a written language in relation to L1 proficiency of a signed language.

In addition to the deaf participants, ten hearing children were assessed on the English sentence comprehension task. These children were included in the study not as a control group *per se*, but only to provide a baseline of how hearing children perform on this task. Our goal was not to draw direct comparisons between the hearing and deaf groups, only to have the hearing group serve as a qualitative indicator of what should be expected in terms of native English sentence reading ability.

## Methods

### Participants

All procedures were approved by The University of Western Ontario Office of Research Ethics. A total of 51 deaf children and adolescents, ages 7;3 to 19;0, were recruited from two Schools for the deaf in southern Ontario. Six were children of deaf parents, and all reported first exposure to ASL before the onset of puberty. Written informed consent was obtained from individuals older than 18 years, or from a parent for all individuals younger than 18 years of age. ASL was the exclusive language of instruction and communication inside and outside the classroom, the one exception being English literacy training, which occurred through reading and writing activities. All students used ASL to communicate and were severe to profoundly deaf (70+ dB hearing thresholds).

A group of ten hearing children, ages 8;02 to 8;11 were also included in the study. These children were recruited through The University of Western Ontario Participant Pool and were assessed in our laboratory.

### Procedures

The deaf children were tested individually in a private room in their school, over two sessions. Session one consisted of the ASL and English reading tasks, and took approximately 40 minutes to complete. Session two consisted of a hearing assessment and a nonverbal intelligence test. Note that this study was part of a larger research project that investigated the factors influencing English and ASL proficiency in deaf children, and included some additional tasks intended to test phonological and single-word knowledge in ASL and English. Those tasks did not involve sentence recognition skills and so data from them are not reported in the present study.

Stimuli for the language tasks in session one were presented via a 12-inch Macintosh PowerBook or a 13-inch MacBook computer placed directly in front of the seated participant. The researcher sat next to the participant and recorded responses on prepared score sheets. As described below, all language tasks were receptive in nature, and therefore the experimenter was not required to interpret children's signs. However the experimenter was a fluent (hearing) signer and was able to answer any questions that arose during the sessions. Sequence of task presentation was held constant across participants, in the order indicated below.

#### ASL assessment

Three receptive sign language proficiency tasks were administered. We used receptive ASL tasks to maximize comparability to written English comprehension, which is itself a receptive task. Standardized language measures exist for both ASL [27] and English [28]. However, the following tasks were used here since the present experiment was part of a larger study that selectively assessed specific aspects of ASL and English proficiency in a way that was matched across both languages. This is somewhat harder to do using standardized tests, which tend to be quite lengthy and/or conflate different aspects of processing into a single score.

In the *ASL Vocabulary Task*, participants saw four pictures (one target picture and three distractors) arranged into each corner of the computer screen. A video clip appeared at the center of the screen, depicting a native signer producing an ASL sign. The participant was asked to point to the picture that correctly matched the sign. The researcher provided feedback on four practice items, 16 test items followed without feedback (Appendix S1 in File S1).

In the *ASL Sign Decision Task*, participants saw pairs of video clips, each depicting a single ASL sign. In each pair, one clip contained a true ASL sign; the other contained a permutation of that true sign making it invalid. Incorrect foils were created by changing either the handshape, point of articulation or movement feature of the valid sign [29]. In this task the participant was asked to point to the correct sign in the pair. The researcher provided feedback on four practice items, 18 test items followed without feedback (Appendix S2 in File S1). One of these items contained a potentially ambiguous sign pair, and was removed from analyses.

In the *ASL Story Comprehension Task*, participants viewed videos of short stories told in ASL, each of which was followed by five multiple-choice comprehension questions also presented in ASL. The stories and questions were adapted by a native deaf signer from items in Form A of the Gray Oral Reading Test version 4 (GORT-4 [30]; items 1, 2, 4, 6 & 8), ordered with respect to story length and difficulty. We chose to adapt an existing English test to ASL only because it provided a set of stories and questions that are known to be age-appropriate and minimize effects of real-world knowledge that can potentially contaminate story comprehension measures. Moreover, the new task did not assess English reading or the comprehension of syntactic structures in ASL, only ASL story comprehension ability. To begin each trial, the signer presented a short story. Signed instructions indicated that the story could not be repeated—participants had only one opportunity to become familiar with each story. At the end of each story, five new ASL clips appeared on screen; at the center was a video of a question pertaining to the story, and at each corner was a video presenting one of four possible answers. The question and possible answers were played in succession thereby allowing participants to see all of the options. Participants responded by pointing to the video depicting the correct answer. Participants were allowed to view the question and the possible answers as many times as they wished. Prior to the test items, participants viewed a practice story complete with questions and potential answers, to ensure that they understood the task. Feedback was provided during the practice story and questions only. The task was terminated early if a participant answered incorrectly to four or more questions on any one story.

Of primary interest in this study was the degree to which overall receptive ASL proficiency is related to English sentence comprehension. To this end, we obtained a composite score of ASL ability by calculating the average proportion of items correct across all three tasks, with all tasks weighted equally. This composite score was then used to group participants into two groups, henceforth skilled and less-skilled signers. Note that a median split was used to divide signers into these separate groups, and therefore the labels are used in a strictly relative sense rather than to suggest an objective judgment of unusually poor signing ability in the less-skilled group. However, since the goal of the study was to examine differences in English syntactic comprehension between the lower and higher ends of the ASL ability, the distinction serves as a useful way to examine these differences.

#### English sentence comprehension

**Figure 1:**
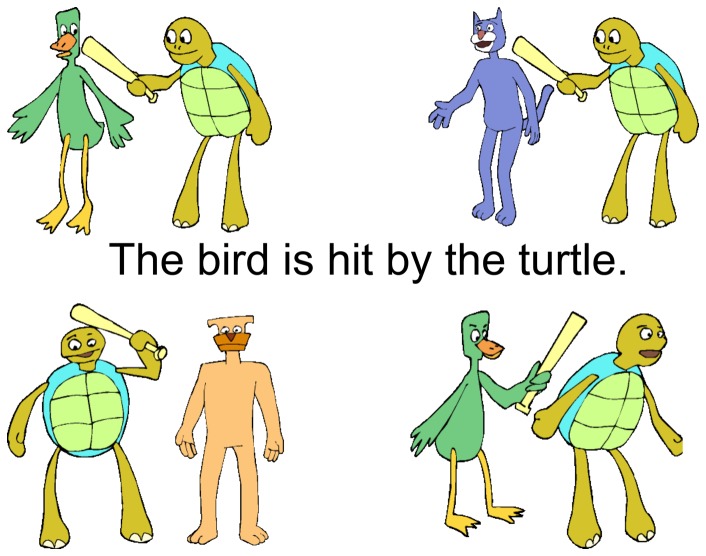
English Sentence Comprehension task. Individuals viewed a written English sentence and chose among four cartoon depictions. Foils included near-miss items (bottom right – agent and patient are reversed) and other-miss items (e.g., top-right and bottom-left picture, which involve actors that are not mentioned in the sentence).

Participants were presented with four pictures (one target picture and three distractors; Figure 1) arranged into each corner of the computer screen. At the same time, a written English sentence appeared across the center of the screen in 44 point sans serif font, without obstructing the pictures. Picture stimuli were cartoon illustrations depicting transitive actions (e.g., washing, pinching, pointing) being performed by humans or other animate creatures (e.g., dogs, cats, turtles). The participant's task was to read the sentence and point to the picture that correctly depicted it. The researcher provided feedback on four practice items, followed by 16 test items presented without feedback. (Appendix S3 in File S1). Both deaf and hearing children participated in this task whereas only the deaf children participated in the previously described ASL measures.

The task was designed to assess comprehension of four types of syntactic constructions: active, passive, pronoun and reflexive pronoun sentences. Examples of each construction were presented four times each throughout the task, for a total of 16 sentences. Each sentence involved a variety of actors and actions including male and female humans, and also common animals. Note that plausibility was maintained for all items, for instance by having all actors variably depicted as subject or object of the verbs, and by depicting animals in a cartoon-like way such that it was possible for them to be pictured either as agent or patient. Sentences were designed so that both proportion correct and proportion of error types could be analyzed. There were two possible types of errors represented on each trial: ‘near-misses’ (at a rate of .25 per trial) and ‘other-misses’ (at a rate of .50 per trial). As explained more fully below and in Figure 1, a ‘near-miss’ involved pointing to a picture that included the mentioned actor(s) and action but did not involve the correct configuration of agent and patient. These errors were considered closer to the correct response than the ‘other miss’ since selection of this picture foil demonstrated recognition of the lexical items in the sentence in spite of incorrectly parsing its syntactic form. In contrast, ‘other-miss’ errors depicted characters, or relationships between the characters, that were unrelated to the target sentence, and thus illustrated a relatively weaker comprehension of the written English sentence.

A near-miss took on two possible forms in this experiment depending on which type of sentence was presented. In the case of active and passive sentences, a near-miss was one in which the patient and the agent were switched (i.e., a word order error). For example, in a sentence that read, “The mother washes the girl” (or in the passive sentences, “The girl is washed by the mother”) the near-miss would be the picture that instead showed the girl washing the mother. Near-misses on the pronoun sentences depicted a misinterpretation of the Binding Principles (i.e., a binding error [18]). For example in a sentence that read, “The mother washes her” the near-miss was a picture that showed the mother washing herself as opposed to the mother washing the girl (vice versa for the reflexive pronoun sentences).

It might be useful to think of the error analysis in terms of educated guesses, which would result in mostly near-misses, versus guessing at random which would result in a proportional mixture of near- and other-misses. Other-misses always depicted more gross departures from the given sentence, and depending on the sentence, included depictions of characters that were not mentioned, characters that represented gender-pronoun mismatches or by pictures that depicted a self-orienting action when non-reflection action is indicated (or vice versa).

#### Hearing and nonverbal intelligence measures

Measures of hearing ability and nonverbal intelligence (NVIQ) were obtained in session two, which took approximately 15 minutes to complete. Both deaf and hearing children also completed the NVIQ portion of session two. Pure-tone hearing thresholds were obtained at 500, 1000, 2000 and 4000 Hz using a standard audiometric procedure [31]. For the purpose of statistical analyses we computed an overall hearing threshold for each child by averaging across all four frequencies. We also averaged across the 500 and 1000 Hz levels, and the 2000 and 4000 Hz levels to obtain low and high frequency thresholds respectively. In our analyses, we operationalized hearing threshold as the lowest dB threshold in the better ear within each frequency range (overall, low, high). NVIQ was assessed using Form A of the Test of Nonverbal Intelligence, version 3 (TONI-3; [32]). This test was specifically designed to eliminate the confound of verbal ability in assessing intelligence, thus it was the ideal intelligence measure for our study. For some of the children, audiometric and TONI-3 scores were available on file at their school. In these cases, the existing scores were used instead, and the children were only assessed on session one (the language measures).

## Results

Our first analysis examined the overall relationship between ASL and English using a Pearson correlation between ASL ability (defined as the composite score on the three ASL tasks) and English reading ability (defined as accuracy on the sentence comprehension task). ASL composite scores positively correlated with average accuracy across the four sentence types, *r* = .74, *p*<.001. This initial finding provided justification for the remaining analyses, which examine the ASL/English relationship in finer detail with special attention paid to the relationship between ASL proficiency and English sentence comprehension.

Deaf children were divided into skilled and less-skilled signer groups (Table 1) using a median split on the ASL composite score. Use of a median split represented a data-driven approach to dividing group into two ability groups. While a median value conceptually divides a sample into two groups, here we obtained somewhat different samples sizes for the two groups due to several individuals' scores falling exactly at the median. We thus included individuals scoring on or above median in the skilled group, and individuals scoring below the median were placed in the less-skilled group. Groups did not differ on age, *t*(49) = 0.99, *p* = .33, or hearing threshold (low frequency threshold: *t*(45) = 0.89, *p* = .38; high frequency threshold: *t*(43) = 0.51, *p* = .62; overall threshold: *t*(43) = 0.90, *p* = .38). The group difference in NVIQ approached significance, *t*(49) = 1.95, *p* = .06; to guard against the possibility that this factor was confounding results, subsequent analyses used ANCOVAs with NVIQ included as the covariate.

**Table 1:** Characteristics of the deaf signer groups.

	Skilled signers (n = 28)	Less-skilled signers (n = 23)
Age (yy;mm)	13;02 (2.70)	12;04 (3.34)
TONI-3 percentile	44.71 (26.60)	30.30 (25.93)
ASL Composite (/58)	43.07 (4.37)	30.95 (4.96)
Vocabulary (/16)	14.68 (1.19)*	10.70 (1.69)
Sign Decision (/17)	16.82 (0.39)*	15.26 (1.81)
Story Comprehension (/25)	11.57 (3.99)*	5.00 (2.32)
Hearing threshold (dB)	92.10 (7.57)	89.91 (8.82)
low frequencies (dB)	88.69 (10.41)	86.14 (8.92)
high frequencies (dB)	95.52 (7.87)	93.97 (11.45)
Number of children born to deaf parents	4	2

*Note*. All values represent group mean, with standard deviation in parentheses.

*Skilled group significantly greater than less-skilled group, *p*<.05.

**Figure 2:**
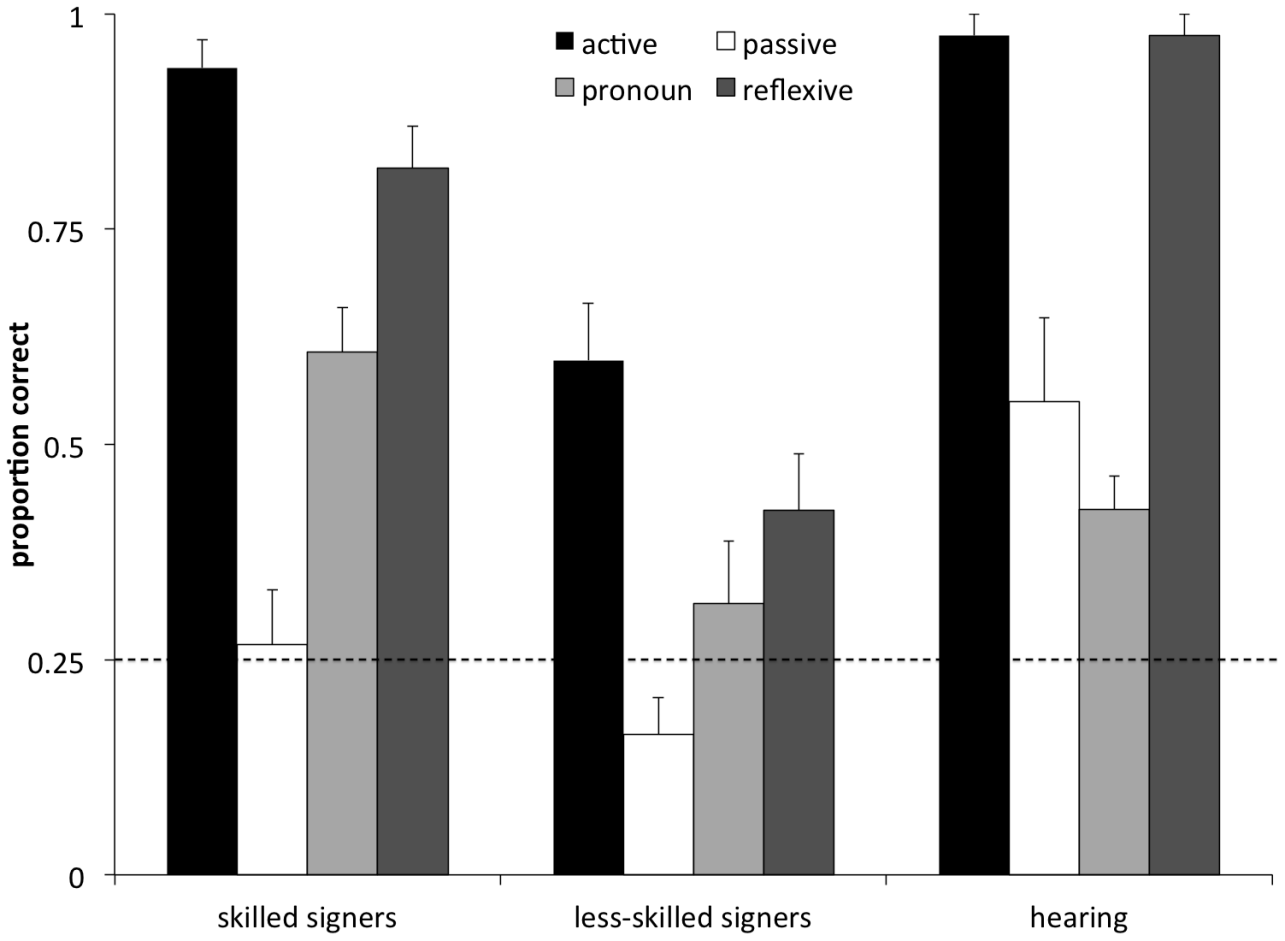
Sentence comprehension scores by group. Proportion of sentences correctly comprehended per group by sentence type. Chance performance equals .25. Error bars indicate standard errors; dotted line indicates chance performance of 25% correct.

Group accuracy on the sentence comprehension task (Figure 2) was compared using a two-way mixed ANCOVA for the effects of group (skilled, less-skilled) and sentence-type (active, passive, pronoun, reflexive pronoun). This revealed main effects of group, *F*(1, 48) = 25.88, *p*<.001, and sentence type, *F*(3, 144) = 11.88, *p*<.001, and a significant interaction, *F*(3, 144) = 2.99, *p*<.05. Post hoc tests revealed that skilled signers outperformed less-skilled signers on active, *F*(1, 166) = 18.39, *p*<.001, pronoun, *F*(1, 166) = 13.58, *p*<.001, and reflexive sentences, *F*(1, 166) = 25.18, *p*<.001, but not on passives, *F*(1, 166) = 1.75, *p* = .19.

Within groups, we examined performance on each sentence type using one-sample t-tests that compared accuracy rates to a chance level of .25. The skilled signers performed significantly above chance on active, *t*(27) = 20.77, *p*<.001, pronoun, *t*(27) = 6.47, *p*<.001, and reflexive pronoun sentences, *t*(27) = 11.94, *p*<.001, but not on the passive sentences, *t*(27) = .28, *p* = .78. The less-skilled group showed a different pattern, performing significantly above chance on actives, *t*(22) = 5.25, *p*<.001, and reflexive pronouns, *t*(22) = 2.65, *p*<.05, but not on pronouns, *t*(22) = .625, *p* = .54, or passive sentences, *t*(22) = −2.01, *p* = .06.

Further, we noted that the skilled group performed more accurately on actives than passives *t*(27) = 9.83, *p*<.001, and more accurately on the reflexives than pronouns *t*(27) = 4.50, *p*<.001. Likewise, the less-skilled signer group performed more accurately on actives than passives *t*(22) = 4.36, *p*<.001, however no accuracy difference existed between the reflexive pronouns and pronouns sentences in this group, *t*(22) = 1.59, *p* = .13.

**Figure 3:**
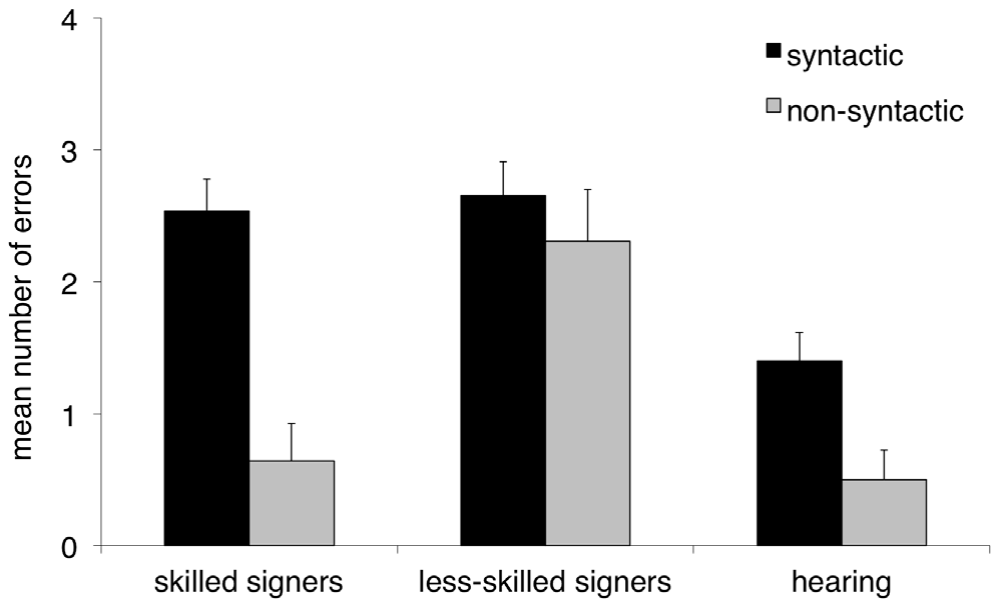
Error analysis for sentence comprehension task – word order trials. Mean number of near-miss and other-miss errors on the word order trials. Error bars indicate standard errors.

**Figure 4:**
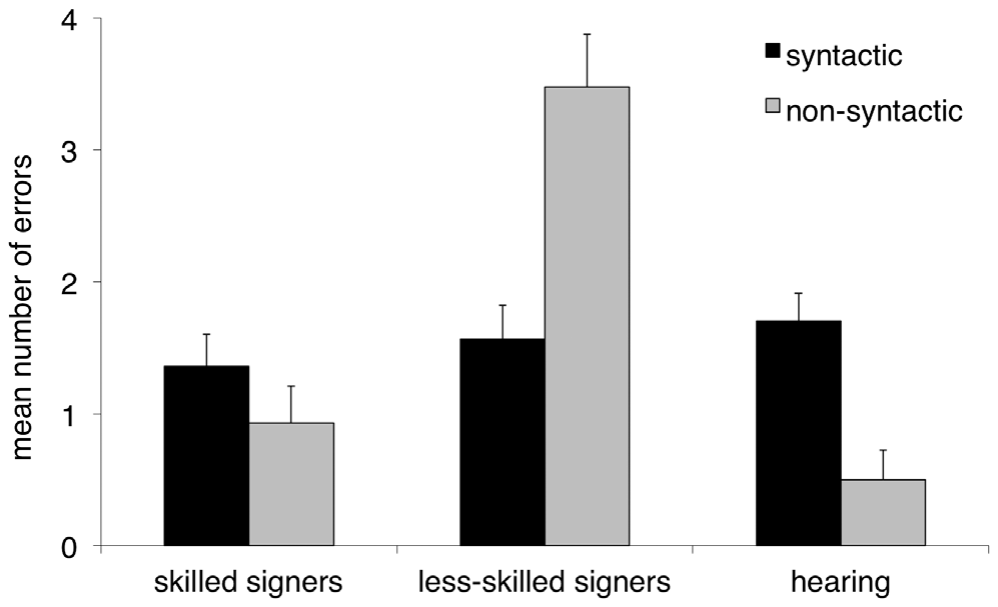
Error analysis for sentence comprehension task – pronoun trials. Mean number of near-miss and other-miss errors on pronoun binding trials. Error bars indicate standard errors.

We also compared the types of errors made on the two trial types (word order, pronoun binding) using two mixed ANCOVAs for the effects of group and error type (near-miss vs. other-miss; Figure 3, 4). On word order trials there was a main effect of group, *F*(1, 48) = 17.58, *p*<.001, and a group by error type interaction, *F*(1, 48) = 10.89, *p*<.05. There was no main effect of error type, *F*(1,48) = .27, *ns*. Post hoc tests revealed that signers in the less-skilled group made significantly more other-miss errors than skilled signers, *F*(1, 97) = 24.15, *p*<.001, on word order trials. No group difference was found with respect to near-miss errors, *F*(1, 97) = .119, *ns*. On pronoun binding trials there was a main effect of group, *F*(1, 48) = 19.93, *p*<.001, and a group by error type interaction, *F*(1, 48) = 12.09, *p*<.01. There was no main effect of error type, *F*(1,48) = 1.40, *ns*. Post hoc tests again revealed fewer other-miss errors in skilled signers, *F*(1, 97) = 36.98, *p*<.001. As above, no group difference was found with respect to the near-miss errors on the pronoun binding sentences, *F*(1, 97) = .246, *ns*.

### Hearing Children

As noted above, deaf children showed better performance on actives vs. passives, and reflexive pronouns vs. pronouns. To summarize, the signers in the high-skilled group performed above chance on all English sentences types except for passives, whereas the signers in the low-skilled group performed above chance on the actives and reflexive pronouns only. Of interest was how these patterns compared to the typical progression of syntactic development shown by first language hearing learners of English. Importantly, this group did not differ with respect to NVIQ from either of the signer groups, *F*(2, 60) = 1.98, *p* = .15. Hearing children showed higher accuracy rates for actives vs. passives, *t*(9) = 4.30, *p*<.01, and reflexive pronouns vs. pronouns, *t*(9) = 11.00, *p*<.001, and performed above chance on all four sentence types, all *p*<.05. They also made significantly more near-miss than other-miss errors on both word order and pronoun binding trials, *t*(9) = 3.09, *p*<.05; *t*(9) = 2.586, *p*<.05, similar to the pattern that what was found in the skilled signers group.

## Discussion

It is well established that deaf individuals are generally delayed in learning to read English, performing on average at a fourth-grade level [10]. Interestingly, across a large number of studies ASL ability has been shown to be the single best predictor of their English reading ability [6]. In light of this, the contribution of the present study was to consider whether how this relationship specifically holds for processing of the syntactic structure of written English, and also whether such effects are best explained as the direct transfer of ASL knowledge to congruent structures in English.

As predicted, there was a strong association between ASL proficiency and English sentence comprehension, marked by differences in sentence reading performance in the skilled vs. lower-skilled group overall. The differences in performance were manifest as both higher accuracy scores across three of the four sentence types (excluding only passives), and as function of the types of errors being committed across trial types. One explanation for these results is that higher-achieving signers have improved single word knowledge of English, and that this alone leads to improved English sentence comprehension. The error analysis seems consistent with this to some extent: the lower-skilled signer group made significantly more other-miss errors, which reflected difficulty in recognizing individual words in a written sentence both on word order and pronoun binding trials. We interpret this as evidence of a general lexical identification difficulty in those individuals. This in itself might explain apparent difficulties in sentence comprehension in that group. Skilled signers on the other hand made proportionally more near-miss errors, indicating that they were able to revert to a strategy of matching written words to the pictures when a syntactic analysis failed. As such, it appears that even when syntax became an obstacle, these better signers were able to fall back on their lexical knowledge in order to select a response that more closely reflected the correct answer. That is, the near-miss errors reflect generally good single-word reading ability, even in the absence of difficulties processing the syntactic form of the sentence. From this we conclude that increased ASL ability supports single word reading.

Indeed, it may be that better ASL proficiency yields better English sentence comprehension reading because of direct cross-language transfer effects in which deaf readers use written English words to access their ASL translation equivalents [33]. On this view, improved ASL ability would predict better English reading ability through the formation of direct links between the two codes. Such a finding would be consistent with the predictions of Cummins' Linguistic Interdependence Hypothesis [14], which proposes that specific L1 knowledge potentiates L2 learning through a process of directly translating lexical features from one language to the next.

That said, the present dataset suggests single word reading ability cannot be the whole story, and thus the effects extend beyond a strict lexical mapping account. If increased ASL ability affects only the lexical level of English reading, then when difficulty of English syntax increases (as in non-reflexive pronouns and passives) the skilled signers should look like the lower-skilled signers in terms of accuracy, because in these sentence structures both lexical identification and syntactic comprehension are required in order to select the correct answer. On a pure mapping account, we should only expect to see increased comprehension in the skilled signer group on sentences where the mapping is transparent (as in actives and reflexives). In fact, we observed skilled signers had stronger performance on the pronoun sentences as well. This suggests that proficiency in ASL promotes the development of word-order knowledge that allows skilled signers to move beyond the one-to-one mapping of lexical items and syntactic structures of L1 to L2. Consistent with the Unified Competition Model our results indicate that L1 proficiency promotes both lexical and syntactic abilities in L2, even across sensory modalities (i.e., signed vs. written language).

That said, we did not find support for increased syntactic abilities on the passive sentences. We are not the first to report passive construction difficulties in young deaf signers [25]. One explanation for this is that deaf children receive very limited exposure to passive structures in English. Thus, while we argue that increased ASL ability relates to increased syntactic proficiency in second language learning, we concede that passives appear to be sufficiently difficult that they defy this explanation. On the other hand we also note that newer work examining sentence processing in deaf adults failed to identify significant differences in reading times for passive versus active sentences [34], suggesting that this represents a delay in development rather than a wholesale failure to master learn these English constructions.

We also examined how hearing children performed on the sentence comprehension task. These children showed the same pattern of performance as deaf individuals: actives were easier than passives, and reflexives were easier than pronouns, suggesting that similar constraints operate on how deaf and hearing children learn syntactic principles in English. Similarly, acquisition of English syntax in deaf readers does not appear to be qualitatively different from how it is learned in hearing readers, and is instead delayed to different extents, largely as a function of their sign ability. Note also that the error pattern of the hearing children resembled that of the skilled signers, but not the lower-skilled signers. This finding further highlights the idea that the skilled signers' English sentence comprehension ability more resembles that of normally hearing children.

Some limitations from the present study are worth mentioning. First, deaf participants were drawn from a relatively wide age band spanning early school years through adolescence. The age range is admittedly not optimal for studying questions of language and reading development given that that both abilities can improve significantly with age. However the population of individuals fitting the criteria for inclusion in this study in the southern/southwestern Ontario areas is already quite small, and additional participants are simply not available in this geographic region. Examining a smaller group of children within a narrower age band would thus have severely limited statistical power in this study. Instead, we chose to obtain the largest sample size possible, rather than limiting the pool of potential participants. This more inclusive strategy also maximized the range of ASL abilities, which was important given our interest in identifying individuals with relatively high vs. low proficiency in this regard. One possibility could have been to divide participants into separate age bands, although preliminary analyses suggested that the patterns of responses across age groups did not yield additional information beyond the typical increase in overall performance with age while limiting statistical power by increasing the number of factors being included in our analyses. Finally, we noted that our two ASL proficiency groups did not differ with respect to age, such that the age ranges were relatively stable across the two proficiency levels. This suggests our strategy of including a wide age range did not confound the findings observed in our study.

We also did not preclude children from the study on the basis of nonverbal difficulties. This raises the possibility that children performed more poorly than expected on certain tasks due to cognitive delays. We did observe a non-significant difference between the skilled and less-skilled signer groups in terms of nonverbal IQ scores. However, we noted that the observed sentence processing differences between groups persisted even when these scores were taken into account statistically using ANCOVAs. Thus, this factor alone seems unlikely to explain the differences we observed.

## Conclusions

We examined the relationship between ASL proficiency and English reading ability specifically with respect to how young deaf readers process written English sentences. We found significant differences between higher- and lower-proficiency signers on sentence comprehension accuracy for active, pronoun and reflexive pronoun sentences in favor of the skilled signers. We also found significant differences in the types of errors committed by each group. Together these findings indicate that the ability to extract lexical and syntactic information from written sentences increases as a function of sign ability. Note that this effect was maintained while holding NVIQ constant. We interpret these results as support for theories suggesting that L2 learning is influenced at both lexical and grammatical levels by L1, even across linguistic modalities. Specifically, ASL proficiency in young signers predicted both English word recognition and English syntactic comprehension abilities.

## Supporting Information

File S1 **Supporting appendices.**
(DOCX)

## References

[1] Beech JR, Harris M (1997) The prelingually deaf young reader: A case of reliance on direct lexical access? J Res Read 20: 105–121.

[2] Transler C, Leybaert J, Gombert J (1999) Do deaf children use phonological syllables as reading units? J Deaf Stud Deaf Educ 4: 124–143. 15579882 10.1093/deafed/4.2.124

[3] Friesen DC, Joanisse MF (2012) Homophone effects in deaf readers: Evidence from lexical decision. Read Writ 25: 375–388.

[4] Hanson VL, Fowler CA (1987) Phonological coding in word reading: Evidence from hearing and deaf readers. Mem Cognit 15: 199–207. 10.3758/bf03197717 3600259

[5] Nielsen DC, Luetke-Stahlman B (2002) Phonological awareness: One key to the reading proficiency of deaf children. Am Ann Deaf 147: 11–19. 10.1353/aad.2012.0213 12448128

[6] Mayberry RI, del Giudice AA, Lieberman AM (2011) Reading achievement in relation to phonological coding and awareness in deaf readers: A meta-analysis. J Deaf Stud Deaf Educ 16: 164–188. 21071623 10.1093/deafed/enq049 PMC3739043

[7] Strong M, Prinz P (1997) A study of the relationship between American Sign Language and English literacy. J Deaf Stud Deaf Educ 2: 37–46. 15579834 10.1093/oxfordjournals.deafed.a014308

[8] Padden C, Ramsey C (1998) Reading ability in signing deaf children. Top Lang Disord 18: 30–46.

[9] Hoffmeister RJ (2000) A piece of the puzzle: ASL and reading comprehension in deaf children. In: Chamberlain C, Morford JP & Mayberry RI, editors. Language acquisition by eye.Mahwah, New Jersey: Lawrence Erlbaum Associates Inc.pp.143–163.

[10] Goldin-Meadow S, Mayberry RI (2001) How do profoundly deaf children learn to read? Learn Disabil Res Pract 16: 222–229.

[11] Mayberry RI, Lock E, Kazmi H (2002) Linguistic ability and early language exposure. Nature 417: 38. 11986658 10.1038/417038a

[12] Chamberlain C, Mayberry RI (2008) American Sign Language syntactic and narrative comprehension in skilled and less skilled readers: Bilingual and bimodal evidence for the linguistic basis of reading. Appl Psycholinguist 29: 367–388.

[13] Mayberry RI, Lock E (2003) Age constraints on first versus second language acquisition: Evidence for linguistic plasticity and epigenesis. Brain and Lang 87: 369–383. 10.1016/s0093-934x(03)00137-8 14642540

[14] Cummins J (1979) Linguistic interdependence and the educational development of bilingual children. Rev Educ Res 49: 222–251.

[15] MacWhinney B (2005) New directions in the competition model. In Tomasello M, Slobin DI, editors. Beyond Nature-Nurture Mahwah, New Jersey: Lawrence Erlbaum Associates, Publishers. pp. 79–110.

[16] MacWhinney B, Bates E (1989) The crosslinguistic study of sentence processing. New York: Cambridge University Press.

[17] Isenhath JO (1990) The linguistics of American Sign Language. Jefferson, NC: McFarland.

[18] Chomsky N (1981) Lectures on government and binding: the Pisa lectures. Berlin: Mouton de Gruyter.

[19] Deutsch W, Koster C, Koster J (1986) What can we learn from children's errors in understanding anaphora? Linguistics 24: 203–225.

[20] Chien YC, Wexler K (1990) Children's knowledge of locality conditions in binding as evidence for the modularity of syntax and pragmatics. Lang Acq 1: 225–295.

[21] van der Lely HKJ, Stollwerck L (1997) Binding theory and grammatical specific language impairment in children. Cognition 62: 245–290. 9187060 10.1016/s0010-0277(96)00783-4

[22] Chen Pichler D (2001) Word order variation and acquisition in American Sign Language. Doctoral Dissertation, University of Connecticut.

[23] Janzen T, O'Dea B, Shaffer B (2001) The construal of events: Passives in American Sign Language. Sign Language Studies 1: 281–310.

[24] Baldie BJ (1976) The acquisition of the passive voice. J Child Lang 3: 331–348.

[25] Power DJ, Quigley SP (1973) Deaf children's acquisition of the passive voice. J Speech Lang Hear Res 16: 5–11. 10.1044/jshr.1601.05 4703838

[26] Vasilyeva M, Huttenlocher J, Waterfall H (2006) Effects of language intervention on syntactic skill levels in preschoolers. Dev Psychol 42: 164–174. 16420126 10.1037/0012-1649.42.1.164

[27] Hoffmeister RJ (1999) American Sign Language Assessment Instrument (ASLAI) Boston University: Center for the Study of Communication & The Deaf.

[28] Semel E, Wiig EH, Secord WA (1995) Clinical evaluation of language fundamentals 3 (CELF-3) San Antonio, TX: The Psychological Corporation.

[29] Klima E, Bellugi U (1979) The signs of language. Cambridge, MA: Harvard University Press.

[30] Wiederholt JL, Bryant BR (2001) Gray oral reading test, version 4. Pearson Education, Inc.

[31] American National Standards Institute (2004) Methods for manual pure-tone threshold audiometry (ANSI S3.21-2004) New York: Author.

[32] Brown L, Sherbenou RJ, Johnsen SK (1997) Test of nonverbal intelligence: A language free measure of cognitive ability. Austin, TX: Pro-Ed, An International Publisher.

[33] Morford JP, Wilkinson E, Villwock A, Piñar P, Kroll JF (2011) When deaf signers read English: Do written words activate their sign translations? Cognition 118: 286–292. 21145047 10.1016/j.cognition.2010.11.006 PMC3034361

[34] Traxler MJ, Corina DO, Morford JP, Hafer S, Hoversten LJ (2014) Deaf readers' response to syntactic complexity: Evidence from self-paced reading. Mem Cognit 42: 97–111. 10.3758/s13421-013-0346-1 PMC3864115 23868696

