# Supplementary material for: Sign Language Ability in Young Deaf Signers Predicts Comprehension of Written Sentences in English
Source: PLoS One. 2014 Feb 28;9(2):e89994. doi: 10.1371/journal.pone.0089994 (PMC3938551; doi:10.1371/journal.pone.0089994)
Supplement: File S1 — Supporting appendices. (DOCX) [file pone.0089994.s001.docx]

Supporting Information for

Sign Language Ability in Young Deaf Signers Predicts
Comprehension of Written Sentences in English

Kathy N. Andrew, Jennifer Hoshooley and Marc F. Joanisse

Appendix S1

ASL Vocabulary Items

Canada, raccoon, king, duck, coach, helicopter, letter, pancake, pineapple, stars, destroy, pink, cheese, snow, sit, ski

Appendix S2

ASL Sign Decision Items

| Target Sign |  | Foil Sign | | |
| --- | --- | --- | --- | --- |
|  |  | Handshape | Point of Articulation | Movement |
| PLAY |  | ‘I love you’ hands | play | play |
| ENCOURAGE |  | encourage | above/below each other | encourage |
| ARRIVE |  | arrive | back of non-dominant hand | arrive |
| FLOWER |  | flower | elbow | flower |
| DISCUSS |  | tip of index on palm | discuss | discuss |
| CAT |  | cat | tip of nose | cat |
| MOTHER |  | mother | index contacts chin | mother |
| TRUST |  | trust | hands side by side | trust |
| RELATIONSHIP |  | ‘touch’ hands | relationship | relationship |
| PERFECT |  | ‘six’ hands | perfect | perfect |
| GUILT |  | guilt | wrong shoulder | guilt |
| STICKY |  | ‘seven’ hands | sticky | sticky |
| LONDON |  | London | wrong shoulder | London |
| BLUE |  | *removed from analysis* | | |
| WONDERFUL |  | wonderful | wonderful | out to sides |
| BRAG |  | brag | temples of head | brag |
| INSTITUTE |  | ‘I love you’ hands | institute | institute |
| FANCY |  | fancy (two-handed) | stomach | fancy |

Appendix S3

English Sentence Comprehension Items

Actives

The mother is washing the girl.

The turtle hits the bird.
The man is pointing at the boy.

The girl is pinching the doctor.

Passives

The bird is hit by the turtle.

The mother is washed by the girl.
The boy is pointed at by the man.

The girl is pinched by the doctor.

Pronouns

The mother washes her.

The doctor pinches her.

The man is pointing at him.

The girl washes her.

Reflexive pronouns

The turtle hits himself.

The mother washes herself.

The boy is pointing at himself.

The girl pinches herself.
